# Growth Media for Mixed Multispecies Oropharyngeal Biofilm Compositions on Silicone

**DOI:** 10.1155/2019/8051270

**Published:** 2019-07-09

**Authors:** Matthias Leonhard, Beata Zatorska, Doris Moser, Berit Schneider-Stickler

**Affiliations:** ^1^Department of Otorhinolaryngology, Medical University of Vienna, Waehringer Gürtel 18-20, 1090 Vienna, Austria; ^2^Department of Cranio-Maxillofacial and Oral Surgery, Medical University of Vienna, Waehringer Gürtel 18-20, 1090 Vienna, Austria

## Abstract

**Aims:**

Microbial colonization of silicone voice prostheses by bacteria and* Candida* species limits the device lifetime of modern voice prostheses in laryngectomized patients. Thus, research focuses on biofilm inhibitive properties of novel materials, coatings, and surface enhancements. Goal of this* in vitro* study was the evaluation of seven commonly used growth media to simulate growth of mixed oropharyngeal species as mesoscale biofilms on prosthetic silicone for future research purposes.

**Methods and Results:**

Yeast Peptone Dextrose medium (YPD), Yeast Nitrogen Base medium (YNB), M199 medium, Spider medium, RPMI 1640 medium, Tryptic Soy Broth (TSB), and Fetal Bovine Serum (FBS) were used to culture combined mixed* Candida* strains and mixed bacterial-fungal compositions on silicone over the period of 22 days. The biofilm surface spread and the microscopic growth showed variations from* in vivo* biofilms depending on the microbial composition and growth medium.

**Conclusion:**

YPD and FBS prove to support continuous* in vitro* growth of mixed bacterial-fungal oropharyngeal biofilms deposits over weeks as needed for longterm* in vitro* testing with oropharyngeal biofilm compositions.

**Significance and Impact of Study:**

The study provides data on culture conditions for mixed multispecies biofilm compositions that can be used for future prosthesis designs.

## 1. Introduction

Standard voice prostheses, such as the Provox and Blom-Singer series, are made of medical grade silicone and represent the gold standard in voice rehabilitation for patients after laryngectomy due to head and neck cancer [1]. The biocompatible and soft material is mandatory for folding and atraumatic insertion of a voice prosthesis into the nonsterile fistula between the trachea and the esophagus [2]. Unfortunately, frequent microbial colonization of the valve mechanism leads to transprosthetic leakage and makes replacement after approximately 2-4 months* in vivo* necessary to avoid aspiration and pneumonia [3]. During swallowing, typically isolated species (*Candida* species, Staphylococci, and Streptococci) from the oral and oropharyngeal microbiome descend to the esophagus and colonize the esophageal prosthesis flange and valve flap [4]. The macroscopically visible mesoscale biofilms evolve over weeks and compromise the valve closure.* Candida albicans* strains have been observed to even infiltrate the silicone [4, 5]. A literature overview of* in vitro* models used to study biofilm formation and inhibition on voice prostheses is displayed in Supplementary Material 1 and illustrates the efforts that have been made to reproduce this microbial colonization process* in vitro* for future research on novel strategies that could inhibit or slow the biofilm formation as well as the variety of different incubation protocols with varying microbial compositions, culture conditions, and growth media. Thus, the comparison of* in vitro* results and the translation of concepts from bench to bedside are complicated and indicate the need of standardization. As* Candida* species have been identified as the main key players in biofilm formation on voice prostheses, early studies preferred growth media for primarily fungal growth [6, 7]. Meanwhile biofilm research shifted to impressive complexity of mixed bacterial and fungal compositions with quorum sensing and bacterial-fungal cross-kingdom interactions that improve organized survival, proliferation, and dissemination [8–10]. Data on systematic evaluation of growth media for specific microbial compositions, such as oropharyngeal biofilms on voice prostheses, is still scarce in literature, although the impact of growth medium on microbial proliferation is already well known. Dynamic microtiter plate models or flow biofilm models mostly use yeast peptone dextrose medium (YPD), RPMI 1640, or Fetal Bovine Serum (FBS) [11–14]. Tryptic Soy Broth (TSB) is reported to generally support growth of oropharyngeal species. M199, Spider Medium, RPMI 1640, and FBS are reported to induce more filamentous growth in Candida spp., which is the more invasive form of proliferation that might lead to material infiltration. Beside YPD, Yeast Nitrogen Base (YNB) is also a standard growth medium widely in use for* Candida* biofilm studies [15–18]. Goal of this* in vitro* study was to culture and assess the mesoscale growth of pure fungal as well as mixed bacterial-fungal biofilm compositions on silicone with seven different, but commonly used growth media (YPD, YNB, M199, Spider Medium, RPMI 1640, TSB, and FBS) in a biofilm model that is intended to generate stable biofilm deposits of oropharyngeal microbes on prosthetic silicone. An optimum growth medium should support combined balanced growth of the microbial compositions and additionally stimulate hypheal proliferation of* Candida* species to match* in vivo* findings on explanted voice prostheses.

## 2. Material and Methods

### 2.1. Preparation of Microbial Strains

Microbial species were collected from explanted dysfunctional voice prostheses of laryngectomized patients, who have been treated at the Department of Otorhinolaryngology of the Medical University of Vienna. The prostheses were sonicated for 10 minutes to remove loose biofilm debris and then vortexed in 5 ml PBS for 3 minutes. The suspended specimens were plated out, isolated, and identified on agar plates using standard microbiological methods. The collection of specimens was stored at −80°C and thawed before further use. Two microbial compositions were prepared to evaluate the growth medium performance with a mixed fungal composition of* Candida albicans, Candida tropicalis, *and* Candida krusei* and with a mixed bacterial-fungal composition of* Candida albicans, Escherichia coli, Streptococcus salivarius*, and* Staphylococcus aureus*.

### 2.2. Selection of Growth Media

Seven of the commonly used growth media for* in vitro* studies with* Candida* biofilm formulation on voice prostheses were prepared according to the description by the respective manufacturer:

- Tryptic Soy Broth (TSB)
- RPMI-1640 Medium 2% glucose (RPMI 1640)
- Yeast extract peptone dextrose (YPD)
- Yeast nitrogen base (YNB)
- Medium 199 (M199)
- Spider Medium
- Fetal Bovine Serum (FBS)

The composition and specification of each growth medium are elucidated in Supplementary Material 2.

### 2.3. Preparation of Microbial Suspensions

Of each* Candida* species single colonies were picked from preincubated Sabouraud-Dextrose agar (37°C for 24 hours, Becton Dickinson, New Jersey, USA), inoculated in 20 ml of YPD, and then incubated at 100 rpm on an orbital shaker for 24 h. The grown* Candida* cultures were centrifuged for 5 minutes and the supernatants were discarded. The remaining cells were washed with PBS (Morphisto, Frankfurt am Main, Germany) three times. The washed planktonic* Candida* cells were used to prepare 1 McFarland standard (equaling 10^7^ cfu ml^−1^) microbial suspension for each* Candida* species. For* Candida* only biofilms, a mixture of 1 ml of each* Candida *species suspension was mixed into one inoculum.

*S. aureus, S. salivarius*,* and E. coli* were picked from preincubated Columbia 5% sheep blood agar (37°C for 24 hours, bioMerieux SA, Marcy l'Etoile, France) and were suspended in PBS to a cell density of 10^7^ cfu ml^−1^. For mixed* Candida*-bacterial biofilms a mixture of 1 ml of each of the prepared suspensions of* Candida albicans, S. aureus, S. salivarius*,* and E. coli* was mixed into one suspension for further inoculation.

### 2.4. Preparation of Silicone Material Samples

Platelets of 8 mm diameter were punched out of medical grade silicone slabs (Websinger, Wolkersdorf, Austria). The platelets were mounted on surgical steel tips in vertical position, autoclaved for 20 minutes at 125°C, and placed sterile in well plates (CellStar Greiner bio-one, Kremsmünster, Austria). Every growth medium was tested using 12 platelets for each biofilm composition.

### 2.5. Multispecies Biofilm Growth

All platelets were precoated at the beginning of the study with FBS for overnight at 37°C. Then one milliliter of each of the both previously prepared inoculums was mixed with 9 milliliters of each growth medium and added to the silicone platelets in the well titer plates. Continuous incubation was performed on an orbital shaker at 150 rpm at 37°C for 22 days, with only a short pause each day for growth media replacement and biofilm growth analysis. Every 24 hours, the used growth media with planktonic free floating cells were removed from the wells. Then, fresh growth media and planktonic cells were replenished. New well titer plates were used every 7 days to remove the effects of biofilm formation on the walls of the wells.

### 2.6. Biofilm Analysis

Every two days the platelets were washed with sterile PBS and photographed top down with standard lighting and magnification (dnt DigiMicro Scale, Drahtlose Nachrichtentechnik Entwicklungs- und Vertriebs GmbH, Germany) to assess the size of mesoscale biofilm deposits. The visible biofilm surface spread was calculated as percentage of the total platelet surface (100%) using software based image analysis (Biofilm Cartographer, Version 2.9, Medical University Vienna, not commercially available) as described previously [19, 20]. The percentages were arcsine transformed to remove the correlation between mean and standard deviation and the data analyzed by a general estimation equation model with an autoregressive correlation structure and growth medium as a group factor and day of measurement as within group factor. Comparisons between the growth media and FBS as a reference growth medium were done by Bonferroni sequential tests using IBM SPSS Statistics (version 23, IBM Corporation, New York, United States). Graphs were illustrated using Graphpad Prism software (Version 7.0a, GraphPad Software, Inc., La Jolla, CA 92037, USA). For all comparisons, a p-value <0.05 was chosen as significance level. After 22 days all platelets were submerged in 2,5% glutaraldehyde at 4°C for 24 hours, dehydrated in a series of ethanol solutions ranging from 70% (v/v) ethanol in distilled water to absolute ethanol, and dried chemically with HMDS (Hexamethyldisilazane, Sigma-Aldrich Life Science, St. Louis, USA). Four representative samples were sputtered with gold (Sputter Coater: SC502, Polaron, Fisons Instruments, Surface Science Division, Cambridge, UK) and analyzed for each growth medium and microbial composition with scanning electron microscopy (JSM 6310, JEOL Ltd., Tokyo, Japan).

## 3. Results

**Figure 1:**
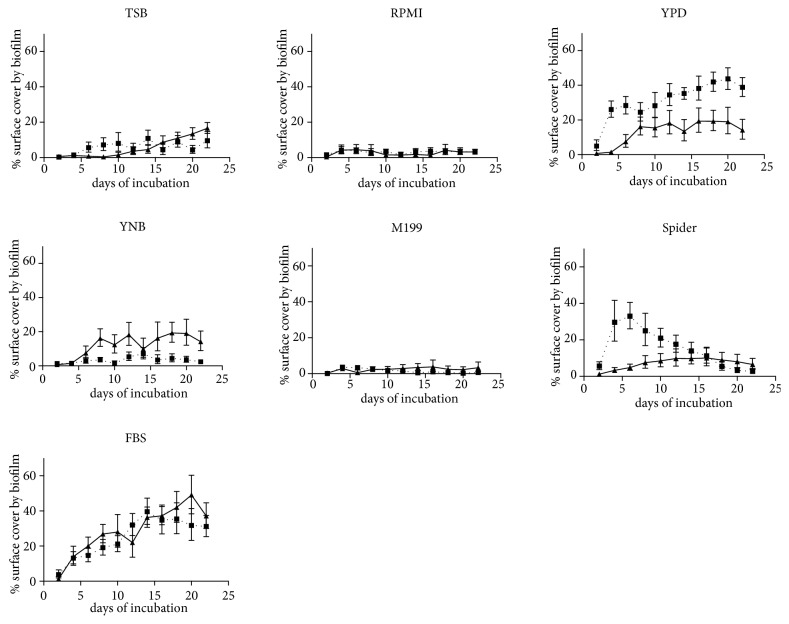
Growth of biofilm deposits on silicone, generated by the tested growth media with two microbial compositions: dotted line:* Candida albicans*,* Candida tropicalis*, and* Candida krusei*. Solid line:* Candida albicans*,* Escherichia coli*,* Streptococcus salivarius*, and* Staphylococcus aureus*. Abbreviations: TSB: Tryptic Soy Broth, YPD: Yeast Peptone Dextrose, YNB: Yeast Nitrogen Base, FBS: Fetal Bovine Serum, and SPIDER: Spider Medium. Error bars show standard deviations of mean values.

**Figure 2:**
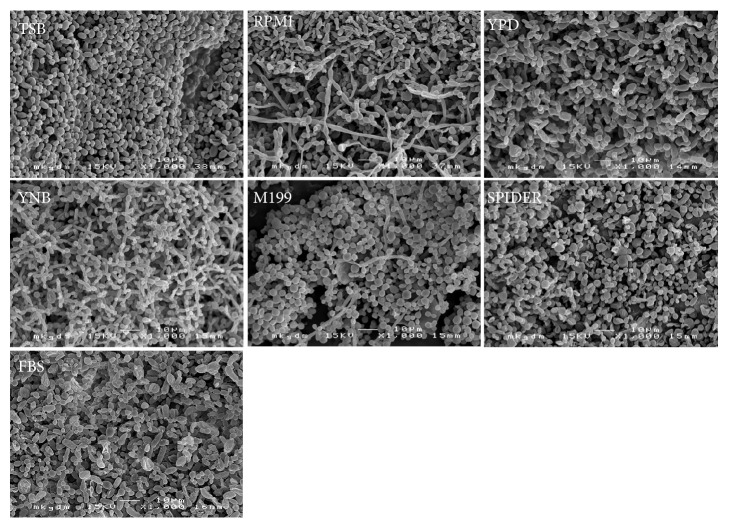
Scanning electron micrographies of biofilm structures of a mixed trispecies biofilm composition of* Candida albicans, Candida tropicalis*, and* Candida krusei* grown in Tryptic Soy Broth (TSB), RPMI 1640 medium (RPMI), Yeast Peptone Dextrose medium (YPD), Yeast Nitrogen Base medium (YNB), M199 medium (M199), Spider medium (SPIDER), and Fetal Bovine Serum (FBS) after 22 days of incubation.

**Figure 3:**
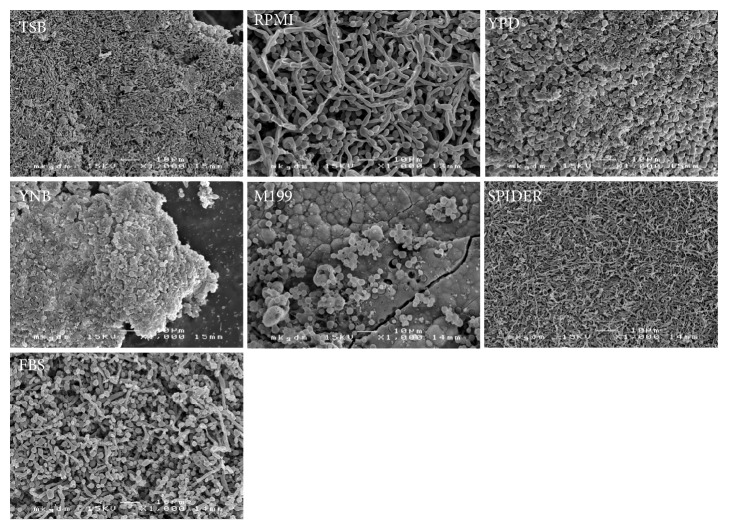
Scanning electron micrographies of biofilm structures of a mixed multispecies biofilm composition of* Candida albicans, Escherichia coli, Streptococcus salivarius*, and* Staphylococcus aureus* grown in Tryptic Soy Broth (TSB), RPMI 1640 medium (RPMI), Yeast Peptone Dextrose medium (YPD), Yeast Nitrogen Base medium (YNB), M199 medium (M199), Spider medium (SPIDER), and Fetal Bovine Serum (FBS) after 22 days of incubation.

The performance of the growth media was evaluated in regard to their ability to generate macroscopically visible biofilm deposits and to produce microscopic biofilm structures. Growth kinetics are illustrated as means of repeated measurements over time in Figure 1. Microscopic images of the resulting predominant growth forms on the examined platelets are displayed in Figure 2 (*Candida* only biofilms) and Figure 3 (mixed bacterial-fungal biofilms). Microscopic key morphologies, such as microbial density, presence of hyphae, and a balance of bacterial and fungal cells, are summarized in Table 1.

### 3.1. Candida Only Biofilm

In absence of bacteria, all tested growth media produced typical microscopic yeast biofilm structures. However, hyphae were only present in the growth media RPMI 1640, YPD, Spider medium, and FBS (Figure 2). No sufficient permanent macroscopically visible colonization was achieved with the growth media TSB, RPMI 1640, YNB, and M199. In contrast, YPD, Spider medium, and FBS supported the growth of visible biofilm mass up to about 30% of the total surfaces of the platelets. However, only YPD and FBS produced a stable and increasing biofilm cover over the whole observation period. Spider medium did not support a permanent biofilm cover, but a peak of about 30% of the total surfaces of the platelets at day 4 of incubation, followed by a slow decline of the mean biofilm deposit sizes.

### 3.2. Mixed Bacterial and Fungal Biofilm

The mixed bacterial-fungal biofilm grew as a permanent, but moderate macroscopic biofilm spread of about 10-20% of the total platelet surfaces in YPD, YNB, and Spider medium. FBS supported an increasing biofilm formation of up to even 40% surface area. RPMI 1640, M199, and TSB showed insufficient permanent surface biofilm colonization, the latter with a slow increase after 12 days of incubation, which proved to be mainly due to bacterial overgrowth in scanning electron microscopy (Figure 3). The presence of fungal hyphae beside budded yeast forms and in coexistence with bacteria was assessed in RPMI 1640, YPD, and FBS.

## 4. Discussion

*In vitro* testing of biofilm inhibitive materials, coatings, and surface structures is an essential step in the development of novel voice prosthesis materials and designs. While most* in vitro* studies focus on single or dual species biofilms and often address the early phases of biofilm formation up to only few hours or days, it is obvious that for voice prostheses more complex bacterial-fungal multispecies biofilm models with extended observation times are needed to benchmark durable antimicrobial efficacy [7, 21–25]. It is also evident that material degradation, hypheal infiltration, and overgrowth of valve mechanisms are a matter of further progressed phases of biofilm colonization that require weeks of incubation* in vivo* before being observed [26]. Antimicrobial material enhancements for voice prostheses should inhibit or slow the formation of mesoscale biofilm deposits and remain effective over time. In this* in vitro* study, the applied biofilm model has been specifically developed to simulate long-term biofilm formation by multiple species similar to* in vivo* biofilm deposits on silicone. However, the tested growth media showed differences in regard to production of visible biofilm surface deposits and microscopic biofilm structures of* Candida *only biofilms and mixed bacterial-fungal biofilms. Thus, preevaluation of growth media is recommended for establishing in vitro biofilm models with specific mixed microbial compositions such as oropharyngeal biofilms. In this well titer plate model, YPD and FBS growth media seem to support such biofilm compositions in long-term studies. Both growth media continuously increase biofilm mass on silicone over 22 days with stimulation of hyphae within a balanced bacterial-fungal growth. However, YPD seems to even better support the proliferation of pure* Candida* biofilms, as the mixed bacterial-fungal composition formed less surface biofilm spread in this growth medium. In comparison, FBS supports the growth of both microbial compositions to a similar degree (Figure 1). With consideration of microscopical results, the low standard deviations of the repeated measurements of the growth curves (coefficients of variation <1) indicate a sufficient methodical precision to assess the performance of each growth medium and microbial composition for in vitro-testing. Thus, a rapid screening of materials and coatings for durable biofilm inhibitive properties is possible and the results could be adopted in similar experimental setups. The results indicate that in vitro growth of even more complex bacterial-fungal compositions might be improved by combination of standard growth media. Another key advantage of well titer plate models compared to open flow models is that the overall amounts of growth media could be reduced to 200 ml of each growth medium for 22 days, which makes it practical and less expensive even for long-term studies using quite expensive FBS.

Methodical limitations of this study are the only 2D assessment of growth of biofilm deposits over time and that microscopically thin biofilm layers could not be detected. Advantage is the evaluation of living biofilm over time. Other methods of quantification, such as MTT, XTT assays or measurement of dry weight, require staining, mortification of cells, or destruction of the biofilm structure and therefore cannot be used for continuous monitoring, while the use of optical coherence tomography requires expensive additional equipment, which is not always available [27, 28].

In summary, pretesting of growth media proves essential for establishing* in vitro* models with mixed bacterial-fungal multispecies compositions and testing standards should be demanded to improve comparison of* in vitro* study results. The growth media YPD and FBS can be recommended for long-term generation of mixed oropharyngeal biofilms on silicone and can be used for* in vitro* screening of biofilm inhibitive materials intended for design of future voice prostheses and new technologies to reduce microbial colonization on medical devices.

## Figures and Tables

**Table 1:** 

Microbial composition	*C. albicans, C. tropicalis, C. krusei* (CCC)							*C. albicans, E. coli, S. aureus, S. salivarius* (CESS)						
Growth medium	TSB	RPMI	YPD	YNB	M199	Spider	FBS	TSB	RPMI	YPD	YNB	M199	Spider	FBS
microbial density	+++	++	++	++	+++	+++	+++	+++	++	+++		+	+++	+++

hypheal growth		++	+	+	+		+		+++	+				+

yeast growth	+++	++	++	++	++	+++	+++		++	++	+	+		+++

bacterial growth								+++	+	++	+		+++	++

Overview of identified microscopic morphologies of *in vitro* generated biofilms based on the microscopic evaluation by SEM of 4 platelet surfaces for each microbial composition and growth medium (no presence, + rare presence, ++ moderate presence, and +++ present in all samples).

## Data Availability

The data used to support the findings of this study are included within the article.

## Supplementary Materials

Supplementary 1 Overview of applied biofilm models specifically used for testing of biofilm inhibitive effects on voice prostheses. It illustrates the variety of in vitro protocols with different microbial compositions, growth media, test materials, and incubation times.* Abbreviations*. TSB: Tryptic Soy Broth, YPD: Yeast Peptone Dextrose, YNB: Yeast Nitrogen Base, FBS: Fetal Bovine Serum, SPIDER: Spider Medium, VP: Voice Prosthesis, and PBS: Phosphate Buffered Solution.

Supplementary 2 Composition and specification of growth media.
