# Supplementary material for: Growth Media for Mixed Multispecies Oropharyngeal Biofilm Compositions on Silicone
Source: Biomed Res Int. 2019 Jul 9;2019:8051270. doi: 10.1155/2019/8051270 (PMC6652045; doi:10.1155/2019/8051270)
Supplement: Supplementary 1 — Overview of applied biofilm models specifically used for testing of biofilm inhibitive effects on voice prostheses. It illustrates the variety of in vitro protocols with different microbial compositions, growth media, test materials, and incubation times. Abbreviations. TSB: Tryptic Soy Broth, YPD: Yeast Peptone Dextrose, YNB: Yeast Nitrogen Base, FBS: Fetal Bovine Serum, SPIDER: Spider Medium, VP: Voice Prosthesis, and PBS: Phosphate Buffered Solution. [file 8051270.f1.docx]

**Supplementary material 1:** Overview of applied biofilm models specifically used for testing of biofilm inhibitive effects on voice prostheses. It illustrates the variety of in-vitro protocols with different microbial compostions, growth media, testmaterials and incubation times.
Abbrevations: TSB: Tryptic Soy Broth, YPD: Yeast Peptone Dextrose, YNB: Yeast Nitrogen Base,

FBS: Fetal Bovine Serum, SPIDER: Spider Medium, VP: Voice prosthesis, PBS: Phosphate buffered solution.

| Author | Date | *Species* | Max. number of species in biofilm | Growth medium | Max. time of biofilm incubation | Test material | Applied in vitro model | Agent tested |
| --- | --- | --- | --- | --- | --- | --- | --- | --- |
| Galli et al. 2018 [1] | 2018 | *C. albicans C. glabrata C. krusei* | 3 | YPD | 24-48 hours | Provox 2 Provox Activalve | Well titer plate model | Silicone Teflon |
| Leonhard et al. 2018 [2] | 2018 | *C. albicans C. tropicalis L. gasseri S. salivarius S. aureus R. dentocariosa* | 6 species | 30% FBS +  70% YPD | 22 days | Provox 2  Provox Vega  Phonax  Provox Activalve  Blom Singer Advantage | Well titer plate model | Silicone  Teflon  Silver oxide |
| Tan et al. 2016 [3] | 2016 | *C. albicans*  *C .tropicalis*  *L. gasseri*  *S. epidermidis*  *S. salivarius*  *R. dentocariosa* | 6 species | YPD | 22 days | Medical grade silicone | Well titer plate model  daily reseeding | Carboxymethyl Chitosan |
| Wannemuehler et al. 2016 [4] | 2016 | *C. albcans*  *C.tropicalis*  *S. salivarius*  *R.dentocariosa*  *S. aureus*  *S. epidermidis* | 6 species | 50% BHI + 50% YPD | 5 days | Explanted Provox 2 VP | Modified Robbins Device  3x per day perfusion  with growth medium | Vibratory stimulus |
| Van der Mei et al. 2014 [5] | 2014 | *C. albicans*  *C. tropicalis*  *mixed with*  *S. aureus*  *S. salivarius*  *S. epidermidis*  *R. dentocariosa*  *L. casei*  *L. acidophilus*  *L. crispatus* | 2 species | 30% BHI + 70% defined  yeast medium | 8 days | Silicone tubes | 3x per day perfusion  with growth medium | Co-incubation with  bacterial strains |
| Leonhard et al. 2013  [6] | 2013 | *C. albicans*  *S. salivarius* | 2 species | RPMI 1640 | 140 days | Medical grade silicone | Well titer plate model  reseeding every 48 hours | In vitro longterm biofilm culturing  material infiltration |
| Ferreira et al. 2013 [7] | 2013 | *E. coli* | single species biofilm | Lysogeny Broth | 24 hours | surface modified  Silicone (Sylgard 184) | Incubation on agar plate | 2-hydroxyethilmethacrylate (HEMA)  and methacrylic acid (MAA) |
| Bandara et al. 2010  [8] | 2010 | *C. albicans*  *C. parapsilosis*  *C. tropicalis*  *C. dublinensis*  *C. glabrata*  *C. krusei*  *P. aeruginosa* | 2 species | TSB | 48 hours | Polystyrene | Well titer plate model | Co-incubation with  *P. aeruginosa* |
| Bandara et al. 2009 [9] | 2009 | *C. albicans*  *C. glabrata*  *C. tropicalis*  *C. parapsilosis*  *C. krusei*  *C. dublinensis*  *E. coli* | 2 species | TSB | 48 hours | Polystyrene | well titer plate model | Co-incubation with  *E.coli* |
| Rodrigues et al. 2006 [10] | 2006 | *S.epidermidis*  *S.salivarius*  *C.albicans*  *C. tropicalis*  *R. dentocariosa*  *S. aureus* | single species biofilm | PBS | Adhesion after 4 hours  Detachment after 1,5 hours | Medical grade silicone | flow chamber | Rhamnolipids |
| Thein et al. 2006 [11] | 2006 | *A. israelii*  *L. acidophilus*  *S. mutans*  *S. intermedius*  *P. nigrescens*  *P. gingivalis*  *P. aeruginosa*  *E. coli*  *C. albicans* | 2 species | YNB | 48 hours | Polystyrene | Well titer plate model | Co-incubation with  oral bacteria |
| Oosterhof et al. 2006 [12] | 2006 | *C. albicans*  *C. tropicalis*  *S. aureus*  *S. epidermidis*  *S. salivarius*  *R. dentocariosa* | 6 species | 30% BHI + 70% defined  yeast medium | 8 days | Groningen Button VP | Modified Robbins Device  3x per day perfusion  with growth medium | Quartery ammonium silane coating |
| Schwandt et al. 2005 [13] | 2005 | *C. albicans*  *C. tropicalis*  *S. aureus*  *S. epidermidis*  *S. salivarius*  *R. dentocariosa* | 6 species | 30% BHI + 70% defined  yeast medium | 7 days | Provox 2 VP | Modified Robbins Device  3x per day perfusion  with growth medium | Diary products |
| Oosterhof et al. 2005 [14] | 2005 | *C. albicans*  *C. tropicalis*  *S. aureus*  *S. epidermidis*  *S. salivarius*  *R. dentocariosa* | 6 species | 30% BHI + 70% defined  yeast medium | 8 days | Groningen Button VP | Modified Robbins Device  3x per day perfusion  with growth medium | Leakage susceptibility |
| Schwandt et al. 2004  [15] | 2004 | *C. albicans*  *C .tropicalis*  *S. aureus*  *S. epidermidis*  *S. salivarius*  *R. dentocariosa* | 6 species | 30% BHI + 70% defined  yeast medium | 7 days | Groningen Button VP  Provox 2 VP | Modified Robbins Device  3x per day perfusion  with growth medium | N-acetylcysteine  Buttermilk  Yakult |
| Adam et al. 2002 [16] | 2002 | *S. epidermidis*  *C. albicans* | 2 species | TSB | 48 hours | Polyvinyl chloride discs | Well titer plate model | Co-incubation with  *S. epidermidis* |
| Gottenbros et al. 2002 [17] | 2002 | *S. aureus S. epidermidis P. aeruginosa E. coli* | single species biofilm | BHI broth, PBS | 1 hour | Medical grade silicone | parallel flow chamber and in vivo model | Argon plasma treated silicone |
| Free et al. 2001 [18] | 2001 | *Frozen rinses from*  *explanted VPs* | not quantified | 30% BHI + 70% defined  yeast medium | 15 days | Groningen Button VP | Modified Robbins Device | Influence of probiotics |
| Arweiler-Harbeck et al. 2001 [19] | 2001 | *Candida wildtype from VPs* | single species biofilm | Sheep blood agar | 7 days | Provox 1 VP |  | Metal coatings of silicone (Al, Au,Ti) |
| Dijk et al. 2000 [20] | 2000 | *Frozen rinses from*  *explanted VPs* | not quantified | 30% BHI + 70% defined  yeast medium | 9 days | Groningen Button VP | Modified Robbins Device | Palladium/tin salt coating |
| Everaert et al. 1999 [21] | 1999 | *Microbiome of 18 patients* | not quantified | in vivo | 8 weeks | Groningen Button VP |  | Perfluoro-alkylsiloxane-modified VP |
| Everaert et al. 1998 [22] | 1998 | *C. albicans*  *C. tropicalis*  *S. epidermidis* | single species biofilm | defined yeast medium  and PBS | 14 days | Silicon rubber | Modified Robbins Device  and flow chamber | Argon plasma treated silicone |

References:

1. Galli J, Calò L, Meucci D, Giuliani M, Lucidi D, Paludetti G, Torelli R, Sanguinetti M, Parrilla C. Biofilm in voice prosthesis: A prospective cohort study and laboratory tests using sonication and SEM analysis. Clin Otolaryngol. John Wiley & Sons, Ltd (10.1111); 2018 Oct;43:1260–1265.

2. Leonhard M, Zatorska B, Tan Y, Moser D, Schneider-Stickler B. In vitro biofilm growth on modern voice prostheses. Head Neck. 2018 Jan;40:763–769.

3. Tan Y, Leonhard M, Moser D, Ma S, Schneider-Stickler B. Long-term antibiofilm activity of carboxymethyl chitosan on mixed biofilm on silicone. Laryngoscope. 2016 Dec;126:E404–E408.

4. Wannemuehler TJ, Lobo BC, Johnson JD, Deig CR, Ting JY, Gregory RL. Vibratory stimulus reduces in vitro biofilm formation on tracheoesophageal voice prostheses. Laryngoscope. 2nd ed. 2016 Dec;126:2752–2757.

5. van der Mei HC, Buijssen KJDA, van der Laan BFAM, Ovchinnikova E, Geertsema-Doornbusch GI, Atema-Smit J, van de Belt-Gritter B, Busscher HJ. Voice prosthetic biofilm formation and Candida morphogenic conversions in absence and presence of different bacterial strains and species on silicone-rubber. Coenye T, editor. PLoS ONE. 2014;9:e104508.

6. Leonhard M, Tobudic S, Moser D, Zatorska B, Bigenzahn W, Schneider-Stickler B. Growth kinetics of candida biofilm on medical polymers: a long-term in vitro study. Laryngoscope. 2013 Mar;123:732–737.

7. Ferreira P, Carvalho Á, Correia TR, Antunes BP, Correia IJ, Alves P. Functionalization of polydimethylsiloxane membranes to be used in the production of voice prostheses. Science and Technology of Advanced Materials. Taylor & Francis; 2013 Sep;14:055006.

8. Bandara HMHN, Yau JYY, Watt RM, Jin LJ, Samaranayake LP. Pseudomonas aeruginosa inhibits in-vitro Candida biofilm development. BMC Microbiol. 2010;10:125.

9. Bandara HMHN, Yau JYY, Watt RM, Jin LJ, Samaranayake LP. Escherichia coli and its lipopolysaccharide modulate in vitro Candida biofilm formation. Journal of Medical Microbiology. 2009 Nov;58:1623–1631.

10. Rodrigues LR, Banat IM, Mei HC, Teixeira JA, Oliveira R. Interference in adhesion of bacteria and yeasts isolated from explanted voice prostheses to silicone rubber by rhamnolipid biosurfactants. J Appl Microbiol. 2006 Mar;100:470–480.

11. Thein ZM, Samaranayake YH, Samaranayake LP. Effect of oral bacteria on growth and survival of Candida albicans biofilms. Arch Oral Biol. 2006 Aug;51:672–680.

12. Oosterhof JJH, Buijssen KJDA, Busscher HJ, van der Laan BFAM, van der Mei HC. Effects of quaternary ammonium silane coatings on mixed fungal and bacterial biofilms on tracheoesophageal shunt prostheses. Appl Environ Microbiol. 2006 May;72:3673–3677.

13. Schwandt LQ, van Weissenbruch R, van der Mei HC, Busscher HJ, Albers FWJ. Effect of dairy products on the lifetime of Provox2 voice prostheses in vitro and in vivo. Head Neck. 2005 Jun;27:471–477.

14. Oosterhof JJH, van der Mei HC, Busscher HJ, Free RH, Kaper HJ, van Weissenbruch R, Albers FWJ. In vitro leakage susceptibility of tracheoesophageal shunt prostheses in the absence and presence of a biofilm. J. Biomed. Mater. Res. Part B Appl. Biomater. 2005 Apr;73:23–28.

15. Schwandt LQ, van Weissenbruch R, Stokroos I, van der Mei HC, Busscher HJ, Albers FWJ. Prevention of biofilm formation by dairy products and N-acetylcysteine on voice prostheses in an artificial throat. Acta Otolaryngol. 2004 Aug;124:726–731.

16. Adam B, Baillie GS, Douglas LJ. Mixed species biofilms of Candida albicans and Staphylococcus epidermidis. Journal of Medical Microbiology. 2002 Apr;51:344–349.

17. Gottenbos B, van der Mei HC, Klatter F, Nieuwenhuis P, Busscher HJ. In vitro and in vivo antimicrobial activity of covalently coupled quaternary ammonium silane coatings on silicone rubber. Biomaterials. 2002 Mar;23:1417–1423.

18. Free RH, Busscher HJ, Elving GJ, van der Mei HC, Van Weissenbruch R, Albers FW. Biofilm formation on voice prostheses: in vitro influence of probiotics. Ann. Otol. Rhinol. Laryngol. 2001 Oct;110:946–951.

19. Arweiler-Harbeck D, Sanders A, Held M, Jerman M, Ehrich H, Jahnke K. Does metal coating improve the durability of silicone voice prostheses? Acta Otolaryngol. 2001 Jul;121:643–646.

20. Dijk F, Westerhof M, Busscher HJ, van Luyn MJ, van der Mei HC. In vitro formation of oropharyngeal biofilms on silicone rubber treated with a palladium/tin salt mixture. J Biomed Mater Res. 2000 Sep;51:408–412.

21. Everaert EP, Mahieu HF, van de Belt-Gritter B, Peeters AJ, Verkerke GJ, van der Mei HC, Busscher HJ. Biofilm formation in vivo on perfluoro-alkylsiloxane-modified voice prostheses. Arch Otolaryngol Head Neck Surg. 1999 Dec;125:1329–1332.

22. Everaert EP, van de Belt-Gritter B, van der Mei HC, Busscher HJ, Verkerke GJ, Dijk F, Mahieu HF, Reitsma A. In vitro and in vivo microbial adhesion and growth on argon plasma-treated silicone rubber voice prostheses. J Mater Sci Mater Med. 1998 Mar;9:147–157.
