# Supplementary material for: Growth Media for Mixed Multispecies Oropharyngeal Biofilm Compositions on Silicone
Source: Biomed Res Int. 2019 Jul 9;2019:8051270. doi: 10.1155/2019/8051270 (PMC6652045; doi:10.1155/2019/8051270)
Supplement: Supplementary 2 — Composition and specification of growth media. [file 8051270.f2.docx]

**Supplementary Material 2:** Composition and specification of growth media

| Medium Number | Medium | Composition | Manufacturer |
| --- | --- | --- | --- |
| 1 | Tryptic Soy Broth (TSB) | TSB: 3 wt/vol % | Oxoid, Hampshire, England |
| 2 | RPMI-1640a | RPMI-1640a: 2,08 wt/vol %  MOPS: 6,91 wt/vol %  Glucose: 3,6 wt/vol % | Sigma-Aldrich Life Science, St. Louis, USA  Sigma-Aldrich Life Science, St. Louis, USA  Merck KGaA, Darmstadt, Germany |
| 3 | Yeast Extract Peptone Dextrose (YPD) | Yeast extract: 0,05 wt/vol %  Glucose: 2 wt/vol %  Peptone water 0,03 wt/vol % | Sigma-Aldrich Life Science, St. Louis, USA  Merck KGaA, Darmstadt, Germany  Oxoid LTD, Hamshire, England |
| 4 | Yeast Nitrogen Base (YNB) | Yeast nitrogen base with ammonium sulphate without dextrose or amino acids: : 0,67 wt/vol %  Glucose 2 wt/vol % | Sigma-Aldrich Life Science, St. Louis, USA  Merck KGaA, Darmstadt, Germany |
| 5 | M199 | M199 with earle salts powder: 0,95 wt/vol %  Tris base: 1,87 wt/vol% adjusted to 7,5 pH | Sigma-Aldrich Life Science, St. Louis, USA  Trizma, Sigma Aldrich Life Science, St. Louis, USA |
| 6 | Spider Medium | Nutrient broth: 0,1 wt/vol %  D-mannitol: 0,1 wt/vol %  K_2_HPO_4_: 0,2 wt/vol % | Sigma-Aldrich Life Science, St. Louis, USA |
| 7 | Fetal Bovine Serum (FBS) | pure | Gibco, Life Technologies Carlsbad, California, USA |
